# Leadership Development Sessions in a Community Health Setting: A Feasibility Evaluation of Participant-Perceived Leadership Preparedness

**DOI:** 10.7759/cureus.114692

**Published:** 2026-08-17

**Authors:** Veda Chanda, Samhitha Buchireddy, Aditi Sawant, Sarika Yarrabolu, Eric Baggerman, Utpal Bhalala

**Affiliations:** 1 Biotechnology, Johns Hopkins University, Baltimore, USA; 2 Pediatrics, Amistad Health, Corpus Christi, USA

**Keywords:** career development, community-oriented primary care, interprofessional education and collaboration, leadership theory, medical leadership

## Abstract

Background

Effective leadership is critical in a community health center (CHC) setting to ensure the delivery of high-quality medical care. There is a lack of sufficient literature on healthcare leadership training within community health settings. Therefore, we aimed to develop and implement a CHC leadership program and to evaluate its feasibility, acceptability, and participant-perceived impact in a community health system.

Methods

In May 2023, we initiated a CHC leadership program at Amistad Community Health Center, with all the healthcare leaders participating in monthly leadership sessions. The sessions, led by an expert, focused on topics such as types of leadership, emotional intelligence, communication skills, conflict management, and time management through didactic and interactive sessions. All 11 participants were surveyed in June 2024 regarding their perceptions of the program using a post-intervention survey. Responses were assessed using the Likert scale (1 = not at all to 5 = completely).

Results

Out of 82% (9/11) healthcare leaders who responded to the survey, 89% (8/9) had >5 years of clinical experience, and 56% (5/9) had >5 years of leadership experience. All quantitative items had a mean score above 4.2/5. Highest mean ratings were observed for professional alignment (4.56), enhanced understanding of leadership concepts (4.56), and preparedness to apply leadership principles (4.56). Qualitative feedback (8/9 respondents) supported the collaborative group format and suggested earlier distribution of materials, more role-play/simulation activities, and expanded participation (e.g., cross-site staff and community guest speakers) in future programs.

Conclusions

This longitudinal, low-cost, well-received, and feasible program in a CHC demonstrated high satisfaction and perceived gains in leadership preparedness among clinical and non-clinical leaders. Future studies should examine whether the program drives measurable improvement in leadership skills or organizational outcomes.

## Introduction

Prior to the groundbreaking Flexner Report of 1910, medical training in the U.S. was predominantly apprenticeship-based, where students learned clinical skills from senior physicians [1]. The Lancet Commission on Medical Education in 2010 revealed “a mismatch between professional competencies and patient needs” [2]. It underscored the need for “leadership and interprofessional education (IPE) to strengthen effective health leadership at the local, national, and global levels” [2]. This “leadership gap” in medical education undermines medical practice and affects community health centers (CHCs) largely [3,4]. Without formal leadership training, physicians are expected to manage change and lead teams in CHCs [4]. Despite this inadequacy, leadership curricula are restricted to residency/fellowship programs, academic/hospital settings, and some community-based primary care, but are limited in CHCs [5].

Awareness of the importance of leadership in healthcare has seen a recent surge, with studies showing a correlation between effective leadership and improved organizational performance, patient satisfaction, teamwork, and team dynamics [3,5]. At A.T. Still University School of Osteopathic Medicine in Arizona, primary care physicians led health equity transformation with formal leadership training by launching the 8-month Primary Care Transformation Executive (PCTE) Fellowship with survey and interview evaluation. It was found that leadership engagement and teamwork increased [6]. Previous literature found that leadership interventions improved individual outcomes, team performance, organizational outcomes, and patient outcomes [5-7]. CHC leadership training shapes team functioning, processes, and culture, which advances interprofessional care coordination and reduces health disparities [6]. However, primary care physicians' leadership potential remains underdeveloped [8]. Primary care serves as the foundation of patient care, serving 75-85% of the general population's annual needs and addressing most healthcare needs [9,10]. Thirty million patients in the US procure primary care from Federally Qualified Health Centers (FQHCs), serving lower-income and underserved patients, underscoring their safety-net role for non-FQHC practices as well [11]. By embedding interprofessional learning in community care, the gap between theoretical knowledge and practical application can be narrowed to build team dynamics (role clarity, communication, teamwork) and promote social responsibility [12]. Trained clinical and non-clinical FQHC/CHC leaders through shared educational experiences would augment the overall functioning of healthcare systems by fostering a culture of collaborative care that spreads across the continuum of care [12].

Primary care in community health care needs leadership training so that quality patient care can be provided to most patients in the U.S. There is a need to develop a low-cost and feasible leadership training program for an interprofessional team of providers in a CHC. The CHC leadership program was explicitly designed to address documented gaps in formal, ongoing leadership training for clinical and non-clinical CHC leaders.

The primary objective of this study was to develop and implement low-cost longitudinal leadership development sessions for interprofessional leaders at an FQHC/CHC and to evaluate their feasibility and acceptability into the working day. Secondary objectives were to assess participant satisfaction, perceived relevance, and self-reported preparedness to apply leadership principles.

This article was previously presented at the 2026 TAMUCC SSCIRA on April 24, 2026.

## Materials and methods

Setting and timeline 

The program was a single-site pilot intervention that took place at Amistad Health, a non-profit FQHC/CHC serving the Coastal Bend region of Texas. The curriculum was offered to CHC leaders from May 2023 through June 2024, and the survey was conducted in June-July 2024. Institutional Review Board approval was waived because it was designated as an internal survey. 

Participants and study size

All 11 healthcare providers who were in leadership positions at Amistad CHC were invited to participate in the leadership training program, representing various departments within Amistad Health. Participants had not undergone prior formal leadership training. This established a shared baseline for the cohort, so the curriculum could begin with foundational leadership topics. Attendance was recorded by the facilitator (E.B.) during each session and was mandatory for all invited leaders. Participants who were unable to attend in person joined the session remotely via an online modality (Appendix 1).

Community health center (CHC) leadership program curriculum structure and components

The sessions were facilitated by the CEO of Amistad (E.B.), who spearheaded the leadership sessions, proposed initiating them at Amistad Health, created the core curriculum, invited participants, and led the individual sessions. Sessions were conducted during lunch periods, lasting on average one hour within the workday (see Table 1). Curricular frameworks and session facilitation style were based on the session facilitator’s personal prior leadership and leadership training experience. 

**Table 1:** General structure of each individual session.

Session component	Time allocated (minutes)
Welcome and Recap	5
Leadership Topic Introduction/Overview	10
Didactic Lecture/Video Component	20
Interactive Discussion/Case Studies	20
Group Reflection and Sharing	10
Action Items and Next Steps	5
Total	70

The instructional delivery followed a hybrid design, incorporating didactic teaching, curated video materials, and interactive case-based discussions. Each session began with preparatory activities to encourage learner involvement and advance learning outcomes. Preparatory activities included completing self-reflection questionnaires, watching assigned leadership videos, and reviewing supplemental reading materials, during which different leadership styles, their advantages, limitations, and situational effectiveness were explored (Appendices 3, 6).

Following this, all sessions included core interactive activities, such as roundtable discussions. The main interactive activity was intended to foster active participation, dialogue, and hands-on implementation of leadership concepts. Participants were encouraged to share and discuss past experiences and reflect directly on their daily practice. Shout-out exercises prompted participants to collectively share and reflect on important leadership traits and characteristics. Intermittent video-based group discussions enabled analysis of leadership concepts through multimedia content, while structured role-play scenarios allowed participants to practice leadership skills in simulated healthcare scenarios. The later sessions regularly incorporated reinforcement activities of previous material at the start of each session. The curriculum framework was developed partially prior to study initiation and finalized following the needs assessment (Appendix 5). Following this, all sessions were delivered in strict adherence to the predefined framework. 

The program was developed and delivered entirely on a volunteer basis. Facilitators received no compensation for developing or leading the sessions, and all instructional content was drawn from freely available open-access materials. The program cost was a working lunch provided at each session, capped at approximately $200 USD per session based on the number of attendees (Appendix 2). 

Survey methodology framework

The key session facilitators (U.B. and E.B.) initially administered an anonymous, post-intervention survey in June 2024 (see Appendix 4). The survey was re-distributed three weeks later to encourage maximum participation. Participants undergoing the CHC leadership program were asked to evaluate their experiences through both quantitative and qualitative responses. A series of Likert-scale (1 = not at all, 5 = completely) questions was used to assess participants’ satisfaction, engagement, and perceived impact on professional development. Participants also rated the format and delivery of training materials and the effectiveness of the trainer on a 1 (Very Poor) to 5 (Excellent) scale.

Participants were invited to rate their agreement with the following statements: the leadership series met my expectations; the leadership series was engaging; I was satisfied with the content covered in the leadership sessions; the topics covered aligned with my professional responsibilities; the sessions were effective in enhancing my understanding of healthcare leadership concepts; and I felt more prepared to apply leadership principles in my daily work after attending these sessions.

The survey instrument was developed by the facilitators (U.B., E.B.) based on the program's primary and secondary objectives (Appendix 4). As the program facilitator (E.B.) co-administered the survey, responses were collected anonymously to reduce potential bias.

Quantitative and qualitative analysis

A statistical analysis of the variables was conducted in Microsoft Excel 365 (Microsoft Corporation, Redmond, Washington, USA). For Likert scale responses, means and standard deviations were computed. Frequencies and percentages were calculated for demographics. The response rate was calculated as the number of survey respondents divided by the total number of program participants. Qualitative thematic analysis was conducted to compile and synthesize recurrent patterns from open-ended participant feedback responses.

## Results

Curriculum content and leadership focus areas

The CHC leadership program was conducted at Amistad Health from May 2023 to June 2024, spanning 13 sessions over 13 months. Attendance for all participants was 100% across all 13 sessions. The curriculum began with a focus on foundational leadership topics during the initial months before transitioning to specialized topics of interest to the participants, such as emotional intelligence, workplace culture, and conflict resolution (see Table 2).

**Table 2:** Overview of the CHC leadership program curriculum. CHC: community health center; EI: emotional Intelligence; PTO: paid time off.

Month	Key topics
February 2023	Review Purpose of Series (2 min). Create Goals of Series (10 min). Leadership Styles Overview and Learning Plan (5 min). Culture Discussion-Viewpoints and visions on culture (30 min). Reflect on Simon Sinek Talk Set Calendar for Future Meetings (5 min)
March 2023	Review February concepts Shout out: Reflections on Establishing trust and cooperation, Roundtable: Share identified examples of where Amistad manager can 'eat last’. Leadership style of the month-Democratic; 1. Identify traits---shout out; 2. When beneficial and when not---shout out. Culture Discussion-Open discussion of '6 Essential Aspects of Workplace Culture'; Round Table: Which are most important to you personally, Pick top two aspects for Amistad and why, As group pick top 2 and discuss applications. Victor Wooten 2016 Commencement excerpt and discussion. Discuss future topics
April 2023	Review February concepts-Shout out: Characteristics of Democratic style leadership, Roundtable: Sharing observation on Amistad culture. Leadership style of the month-Authoritative Leadership; 1. Shout out on characteristics; 2. Video discussion: How is this extreme? How might it be like real life? What techniques were used in the second example of leadership in the video; 3. When beneficial and when not. Reflecting on Conflict Resolution as a Manager-Roundtable: Example of prior conflict we wish we would have handled differently, Discussion: top learnings from Conflict Resolution recommended reading, Roundtable: What of the 5 suggestions in the Conflict Resolution video do you think is most important, Comments on quote from Mike Myatt, Roundtable: one example of Amistad conflict and how best handled, Action Items: shout out---one or two learnings to take forward; Discuss future learning agenda.
May 2023	Review April concepts-Shout out: Characteristics of Authoritative style leadership, Roundtable: Examples of recent conflict seen at Amistad in last month. Leadership style of the month-Laissez-faire Leadership; 1. Shout out on characteristics; 2. Video discussion: How is this extreme? How might it be like real life?; 3. What are some potential benefits? What are some potential negative affects? Reflecting on Emotional Intelligence as a Manager-Roundtable: What think is the most important point on the video, Shout out: One way Emotional Intelligence can contribute to leadership, Discussion: Connection between empathy and social awareness, Shout out: Good example of relationship management, Discussion: Examples where quote by Rosenberg may apply, Roundtable: Amistad example of good or bad emotional intelligence and result
June 2023	Review April concepts-Shout out: Characteristics of Laissez-faire style leadership, Roundtable: What was a way that you came across wrong with an employee. Leadership style of the month-Transactional Leadership; Video discussion: Examples at Amistad, Where effective, where not. Reflecting on Emotional Intelligence as a Manager-Roundtable: What think is the most important point on the video, Shout out: Examples of where Emotional Intelligence is important in leadership, Discussion: Connection between empathy and social awareness, Shout out: Good example of relationship management at Amistad, Discussion: Examples where quote by Rosenberg may apply, Roundtable: Amistad example of good and bad emotional intelligence and result
July 2023	Review June concepts-Shout out: Characteristics of Transactional Leadership, Roundtable: Identify characteristics of high and low EI. Leadership style of the month-Coaching Leadership; Video discussion: Examples at Amistad, Where effective, where not. Reflecting on some key leadership recommendations-Covering the first 7 tips: You're a manager, not a friend; Be consistent in dealing with employees; The buck stops at your desk; Focus on the outcome, not the process; Be able to delegate; Don't pretend you know more than you do; Theory vs Real World
August 2023	Review July concepts-Shout out: Characteristics of Coaching Leadership, Roundtable: Give an example in the last month of where the 'buck stopped' with you. Leadership style of the month-Pacesetter Leadership; Video discussion: Examples at Amistad, Where effective, where not. Reflecting on some key leadership recommendations-Covering the first 7 tips: Just Say No; Share Your Vision; Open up to People; Better to get in trouble for things that you did than the things that you didn't do; Under Promise, Over Deliver; Get it in Writing; Never ask someone to... Reflect on: I see many managers making excuses but very few leaders do!
September 2023	Series break
October 2023	Review August concepts-Shout out: Characteristics of Pacesetter Leadership, Roundtable: Give an example where the 'get it in writing' recommendation has recently applied. Leadership style of the month-Democratic, Authoritative, Laissez-faire, Transactional, Coaching, Pacesetter. Leading, inspiring to the world ahead: Watch and reflect on how this video can apply specifically to our work at Amistad. Role Play-A. Need PTO; B. Disrespect by coworker; C. Angry at management; D. Tardy/Absent

In total, six leadership styles were covered throughout the curriculum: democratic, authoritative, laissez-faire, transactional, coaching, and pacesetter, with one style covered per session. The democratic leadership sub-session emphasized creativity, flexibility, transparency, participation, collaboration, and team input in decision-making. Authoritative leadership centered on building a clear vision around a shared goal, balancing guidance with encouragement, and appropriate contexts where the style is warranted in healthcare practice. Laissez-faire leadership focused on setting goals, making decisions, and resolving problems independently. Transactional leadership explored task fulfillment and reward-based performance management. Coaching leadership examined team development and instructive guidance. Pacesetter leadership trained leaders to set the pace, lead by example, and encourage team members to focus their efforts on the team’s goals.

The cohort attended full sessions on healthcare leadership competencies: emotional intelligence, conflict resolution, and communication skills. During one session, the facilitator (E.B.) led a dedicated session on Emotional intelligence, using Harvard Business School content and real-world applications for relationship management in healthcare settings [13]. The session on conflict resolution was structured around conflict theory, assessment, negotiation and mediation techniques, and application strategies to CHC team practice.

Participant characteristics

The program achieved an 81.8% survey response rate, with nine of eleven participants completing the post-training evaluation survey. Participant composition reflected the inclusive format of the leadership program (see Table 3). Clinicians and allied healthcare staff constituted 22.2% of participants (n = 2). Non-clinical administrative leaders, responsible for clinical workflow and patient care coordination, comprised the remaining 77.8% of participants (n = 7).

**Table 3:** Baseline characteristics of survey participants (N = 9).

Characteristics	Participant number
Position	
Physician	1 (11.1%)
Medical assistant	1 (11.1%)
Non-clinical administrative leader	7 (77.8%)
Patient population	
Adult and pediatric	7 (77.8%)
Pediatric	2 (22.2%)
Years of clinical experience	
1-5 years	1 (11.1%)
5-10 years	3 (33.3%)
10-20 years	3 (33.3%)
>20 years	2 (22.2%)
Years of leadership experience	
1-5 years	4 (44.4%)
5-10 years	3 (33.3%)
10-20 years	2 (22.2%)

Amistad Health offers all-age primary care, with the majority of participants (77.8%, n = 7) providing care to both adult and pediatric populations. Pediatric-focused providers comprised 22.2% of participants (n = 2). This distribution ensured that the CHC leadership program addressed the unique challenges of managing healthcare teams treating diverse age groups and clinical needs.

The participants' professional profiles were evenly distributed across the full range of healthcare experience. The number of participants with 1-5, 5-10, 10-20, and >20 years of healthcare experience was 1 (11.1%), 3 (33.3%), 3 (33.3%), and 2 (22.2%), respectively. Leadership experience among participants varied significantly as well, reflecting a balanced mix of management and supervisory roles. Overall, the number of participants with 1-5, 5-10, and 10-20 years of healthcare experience was 4 (44.4%), 3 (33.3%), and 2 (22.2%), respectively.

Survey results

**Figure 1:**
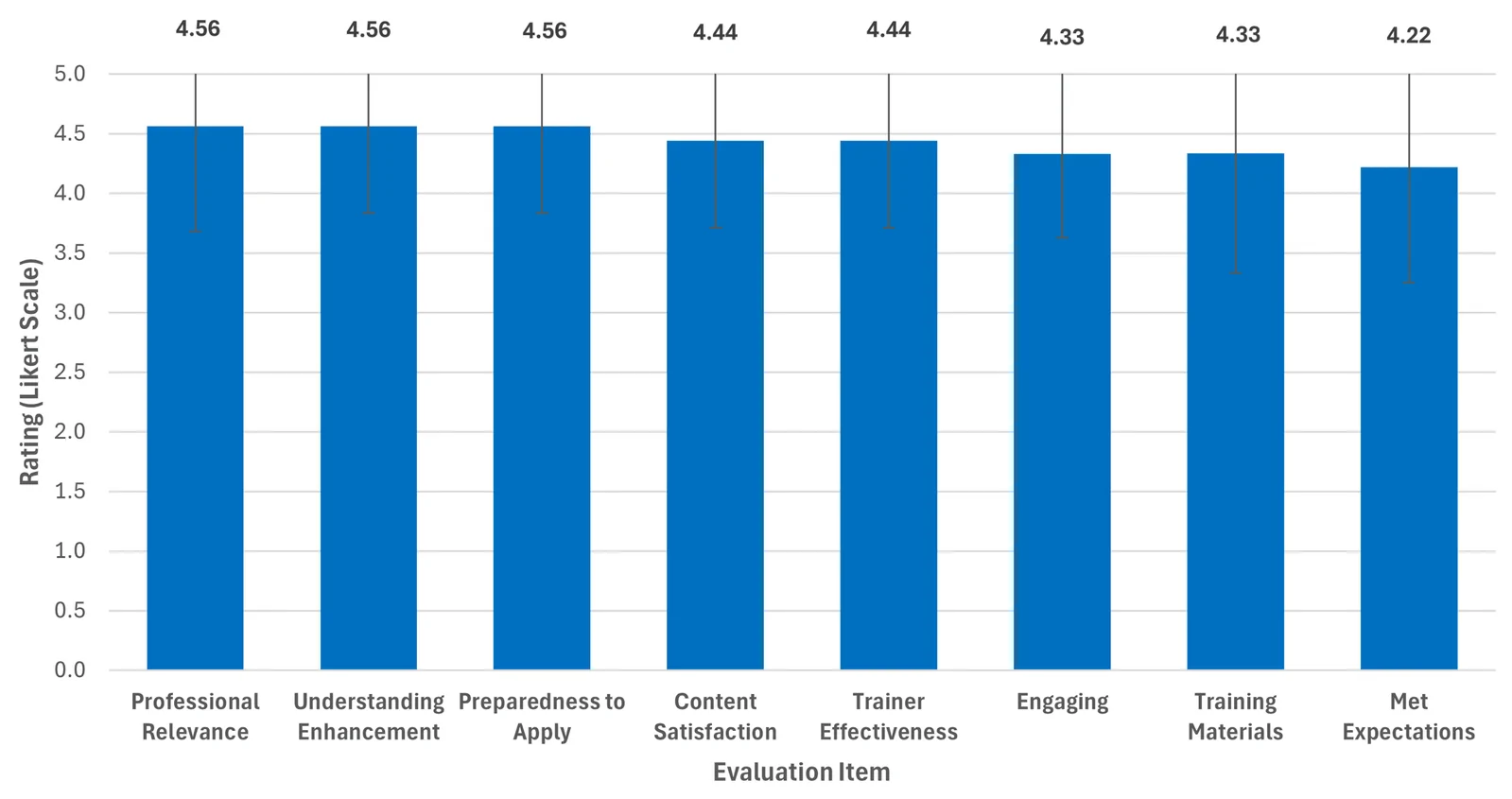
The results from the end-of-program survey. Likert-scale questions assessed participant ratings on professional relevance, enhancement of understanding, preparedness to apply concepts, content satisfaction, training effectiveness, engagement level, expectation fulfillment, and quality of training materials.

Participants perceived the CHC leadership program as highly beneficial at the time of the survey, with all items scoring above 4.2 (see Figure 1). The highest ratings were for professional alignment (mean = 4.56, SD = 0.88), increased understanding of healthcare leadership (mean = 4.56, SD = 0.73), and leadership preparedness (mean = 4.56, SD = 0.73). Participants reported high satisfaction ratings of 4.44 (SD = 0.68) for the program's content and conduct, 4.33 (SD = 0.67) for engagement, and 4.22 (SD = 0.92) for expectation fulfillment. Trainer/facilitator effectiveness was assessed at a mean of 4.44 (SD = 0.68), while the training format and delivery yielded a mean of 4.33 (SD = 1.00). Facilitators and the learning approach received strong endorsement.

In the online survey, eight out of nine respondents (89%) provided qualitative feedback. A thematic analysis of the open-ended responses revealed three primary themes regarding the program's impact and areas for future development:

Theme 1: Value of Team-Oriented Learning and Psychological Safety 

Participants shared overwhelmingly positive impressions, with several rating the program as “outstanding” and expressing interest in future sessions. A major sub-theme was the appreciation for the team-oriented learning environment and engaging discussions. Specifically, one participant emphasized Simon Sinek’s concepts as an effective tool for teaching trust-building and safety within teams [14]. Discussions exploring how good leaders nurture trust and support group behavior were highly valued by the cohort.

Theme 2: Opportunities for Curricular and Administrative Enhancement 

Respondents provided actionable feedback on administrative and structural improvements. Innovative ideas to enhance the curriculum included utilizing varied preparatory work, rotating facilitators, and implementing mid-month follow-ups. To improve efficiency and material comprehension, participants suggested distributing class information and materials prior to the sessions. Additionally, multiple respondents recommended an increased integration of case studies and simulated sessions to improve engagement and the practical application of leadership skills.

Theme 3: Expanding Organizational and Community Impact 

The final theme centered on scaling the program's impact beyond the immediate cohort. Suggestions for broader organizational alignment included inviting management staff from other sites to participate in group meetings. Furthermore, participants recommended integrating community guest speakers to share diverse perspectives and experiences, thereby enriching the leadership context.

## Discussion

Our bespoke CHC leadership program aimed to develop and evaluate the feasibility of a leadership curriculum for CHCs in the United States. We deliberated on major gaps identified in current leadership training due to three main observations: (i) healthcare professionals undertake leadership responsibilities without formal training, (ii) a lack of knowledge regarding effective leadership training designed for CHC-based primary care, and (iii) how IPE should be conducted for leaders to provide “collaborative care in practice” [15]. The study addressed three key questions in support of this objective. First, we sought to identify participants’ preferred training method. Second, we aimed to identify key leadership concepts and topics to incorporate into the curriculum based on the learners’ feedback. Third, we evaluated whether participants perceived the CHC leadership program as having improved their leadership preparedness. These interconnected questions supported the study’s primary goal of designing and testing the feasibility of a leadership curriculum for CHC leaders.

Leadership development initiatives within clinical environments emphasize team-based, discussion-driven learning models. These approaches have shown promising outcomes for both engagement and retention in healthcare settings. The collaborative nature of clinical work and evidence suggests that these approaches enhance clinicians’ collaboration skills, cohesiveness, and willingness to assume leadership roles [9]. A study exploring leadership development in pre-doctoral dental curricula found that participants not only demonstrated significant increases in leadership capability but attributed this increase, in part, to team-based practice management simulations [16]. Such team-based models enhance clinical skills associated with effective collaboration among healthcare professionals. Flexible curricula have also been attributed to increased levels of retention. At an academic health center, physician faculty who completed a structured, yet flexible leadership development program exhibited an annual attrition rate significantly lower than both institutional and national norms, suggesting that leadership training may contribute to faculty retention through individualized development [17]. The relevance of these findings is particularly strong for community health clinics, where staffing shortages and limited professional development resources are ongoing challenges. Team-based and flexible leadership sessions align well with the realities of community health workflows, therefore reducing barriers to participation while improving collaborative leadership.

Our survey was unique due to its wide scope of satisfaction markers, allowing it to assess multiple dimensions of participant experience rather than focusing on just one or two conventional metrics and hence resulting in a more comprehensive evaluation. The high survey response rate in our study may have been attributed to Amistad Health’s small, tight-knit workforce, where individuals were already comfortable collaborating and sharing interpersonal trust. Conducting the survey in a familiar local setting likely fostered a sense of responsibility and accountability, encouraging participants to remain engaged throughout the study and to complete the survey in full. In one study of Hospital Consumer Assessment of Healthcare Providers and Systems (HCAHPS) surveys, the relationship between response rate and reported patient experience scores varied significantly between the smallest and largest hospitals, suggesting response bias or differential engagement in larger settings [18]. From a leadership perspective, these differences are highly relevant. For community health leaders and practitioners, understanding that CHCs may have a higher baseline survey participation but greater staff turnover means that survey strategies must be adapted. Given that staff participation directly affects quality and engagement, leaders should prioritize not only survey completion but also acting on results in ways that improve retention.

Our study found that most participants prefer training methods that involve more follow-ups, preparatory information and work, and inviting other community healthcare staff to participate and speak on their experiences. A survey mean rating of 4.44/5 for trainer/facilitators suggests that the participants found our session methods effective. Several participants described how they would prefer to discuss leadership topics specific to the clinic’s needs, such as evaluating job descriptions. Overall, the participants gave a mean rating of 4.44 out of 5 for the survey question “the topics covered aligned with my professional responsibilities,” suggesting that the discussion topics were beneficial to their specific values and roles.

A systematic review by Sadowski et al. on the effectiveness of leadership training curricula in medical education found that the most effective teaching methods were “small group teaching, mentoring, coaching, and project work” over a longitudinal, consecutive timeline [19]. Our methods aligned with the findings of Sadowski et al., with our use of small-group discussion among 11 staff and mentoring from the session leader and the CEO of Amistad Health (E.B.). However, our methods did not pretest the participants "for learning style and personality characteristics" through Myers-Briggs indicators and feedback, as suggested by Sonnino's systematic review [20]. This could be a potential limitation of our leadership training and is worth considering for future sessions.

In a study on training community healthcare workers (CHWs) at an HIV clinic, the training model was created to build CHW-specific skills, which included “empowering leadership” [21]. The study concluded that the skills developed from such curricula were necessary for community health because they allowed providers to create an organized system that functioned as a safe space that broke down barriers, like stigma and socioeconomic inequality, for their patients [21].

Major national and international bodies, including the Centers for Medicare & Medicaid Services (CMS), the Department of Health and Hospitals (DHH), The Joint Commission, the World Health Organization (WHO), the American Health Information Management Association (AHIMA), and the Interprofessional Education Collaborative (IPEC), endorse IPE [15]. IPE helps generate “an integrated model of collaborative care in practice” [15]. IPE improves teamwork, enhances communication, and positively impacts quality health measures and clinical outcomes [22]. Physicians and nursing professionals have been represented in 87% and 82% of IPE-based curricula in North America, respectively [22]. However, allied health, clerical, administrative, and other non-clinical staff are not sufficiently exposed to IPE [22]. The IPEC describes its central goal as an interprofessional collaborative practice to deliver “safe, high-quality, accessible, and patient-centered care” [23]. We attempted to standardize a strategic, system-level, reproducible, high-quality, and realistic leadership curriculum. Our program convened an interprofessional team for joint training of clinical and non-clinical participants, aligning with IPEC’s goal of supporting the delivery of integrated primary care.

Longitudinal, spaced, multi-session designs deliver stronger, more sustained outcomes [19,24-26]. Few programs combine longitudinal spacing and practice-integrated programs with IPE, highlighting the distinctiveness of our program’s framework. The CHC leadership program lasted 13 months. This surpassed the six-month median duration of similar interventions, with only 15% of programs reaching a full year in systematic reviews [27]. The longitudinal, spaced, multi-session design was intended to support learning “stickiness” through repeated cycles of practice, feedback, and reflection, a mechanism associated in the wider literature with improved retention, practical application, and long-term performance relative to one-off or massed interventions [19,24,26,28]; retention and application were not directly measured in the present study.

Overcoming the pitfall of limited resources, CHCs try to address health inequities, build trust, and treat underserved populations [29]. CHCs continue to offer low-cost, high-quality primary care, despite functioning with inadequate personnel and facing ongoing funding gaps [29,30]. Our program was designed around Amistad Health’s community-driven mission of comprehensive, affordable, community-governed care for underserved patients. It embeds learning in the workplace, explicitly linking leadership development to FQHC-aligned operational pathways intended to help ensure patient needs are met despite barriers to affordable care. A low-cost CHC leadership program of this kind may be readily adopted by similar, resource-limited CHCs, and may support service delivery through interprofessional teams and stakeholder performance. 

Limitations

This study has several important design limitations. First, it used a post-intervention self-assessment survey (Likert scale) with no baseline (pre-intervention) measurement and no objective assessment of leadership competency. Second, given equity concerns from withholding the intervention to eligible participants, the study lacked a control group. Accordingly, improvement cannot be attributed to the program. Third, the single-institution setting and small sample limit precision, external validity, and generalizability. Fourth, as participants belonged to the same organization and the program facilitator both led the sessions and co-administered the survey, social desirability and response bias are possible. We sought to mitigate this potential bias through anonymous data collection. Fifth, the survey instrument was developed internally and was not formally validated or pilot-tested, and qualitative responses were grouped thematically without a formal coding framework. Sixth, no long-term follow-up was conducted, so skill retention and any change in workplace practice or behavior beyond the immediate post-program period are unknown. Finally, causality cannot be inferred from this uncontrolled, post-only design. 

## Conclusions

The CHC leadership program at Amistad Health trained clinical and non-clinical leaders through monthly interactive sessions covering leadership styles, emotional intelligence, communication, conflict management, and time management. Our post-training survey with a high response rate revealed high participant satisfaction across domains such as expectation fulfillment, engagement, and perceived leadership preparedness. Future studies should incorporate pre- and post-assessments, validated leadership-competency instruments, and behavioral or organizational outcome measures to determine whether these perceived gains translate into measurable improvement.

## Appendices

This supplement provides implementation details to support the reproducibility of the CHC leadership development program. It comprises five appendices: session attendance (S1), program cost (S2), learning objectives, instructional materials, and assessment methods (S3), the survey instrument (S4), the needs assessment (S5), and online materials (S6).

Appendix 1: Session attendance summary

Participation in the leadership development series was mandatory for all 11 invited clinical and non-clinical leaders at Amistad Health. Attendance was recorded by the facilitator (E.B.) at the start of every session.

Attendance was 100% for all 13 monthly sessions and for all participants. Participants who were unable to attend a session in person joined the same session synchronously via an online modality, and remote attendance was counted as attendance. No participant withdrew from the program, and no session was missed by any participant.

**Table 4:** Session attendance summary.

Attendance metric	Value	Notes
Sessions delivered	13 of 13 planned	Monthly, May 2023-June 2024
Invited participants	11	All leadership-position staff at the site
Overall attendance rate	100%	All participants, all sessions
Attendance policy	Mandatory	Attendance recorded by the facilitator at each session
Remote attendance option	Available	Synchronous online participation counted as attendance
Withdrawals	0	-

Appendix 2: Program cost

The program was designed, developed, and delivered on a volunteer basis. Facilitators (E.B. and U.B.) received no compensation for curriculum development or for leading the sessions. No protected or bought-out time was allocated for these activities.

The only direct program cost was a working lunch provided at each session, capped at a maximum of approximately US$200 per session to cover attendees. Across 13 sessions, this corresponds to a maximum direct outlay of approximately US$2,600 for the full 13-month program.

The principal indirect cost was participant and facilitator time: approximately 70 minutes per session (see Table 1 of the main manuscript), scheduled during the working lunch periods, so that no clinical sessions were displaced.

**Table 5:** Program cost.

Cost component	Amount	Basis
Facilitator compensation	US$0	Curriculum development and session delivery were entirely voluntary
Instructional materials	US$0	Open-access resources only; itemised and cited in Table 2
Working lunch per session	≤US$200	Catering for attendees at each monthly session
Total direct cost, full program	≤US$2,600	13 sessions × US$200 maximum
Indirect cost (time)	~70 min per participant per session	Scheduled within the working day, largely over lunch periods
External funding	None	No grant, sponsorship, or institutional training budget was used

Appendix 3: Session learning objectives, instructional materials, and assessment methods

CHC Leadership Development Sessions, Amistad Health. Objectives are stated as observable participant outcomes. All assessment was formative and participation-based; no graded or summative assessment was administered.

**Table 6:** Session learning objectives, instructional materials, and assessment methods.

Session	Learning objectives (by the end of the session, participants will be able to:)	Instructional materials	Assessment methods
February 2023 (program launch)	1. Identify their own leadership strengths, development needs, and supervisory challenges through a structured pre-session self-assessment. 2. Contribute to a shared set of desired outcomes and ground rules for the series. 3. Discuss viewpoints on organizational culture and relate concepts of trust and psychological safety from the Simon Sinek talk to their own teams.	• Pre-session needs assessment (six open-ended items on best leadership quality, quality to improve, least-understood aspect of leadership, biggest struggle supervising others, others' struggles working with them, and most important development support Amistad can provide) • Leadership styles overview and written learning plan • Simon Sinek video on trust and safety in teams (12 min)	• Written responses to the six pre-session questions, submitted in advance and collated confidentially by the facilitator • Facilitator observation of participation in goal-setting and culture discussion
March 2023	1. Recall February concepts on establishing trust and cooperation, and identify situations in which an Amistad manager can “eat last.” 2. Identify the traits of democratic leadership and state when the style is and is not beneficial. 3. Rank the six essential aspects of workplace culture individually and as a group, and discuss their application at Amistad.	• “6 Essential Aspects of Workplace Culture” video • Written summary of five key features of democratic leadership (encouraging creativity and collaboration, seeking feedback, maintaining a team-player attitude, allowing flexibility, prioritizing transparency and communication) • Victor Wooten 2016 commencement address excerpt	• Shout-out recall of February concepts • Roundtable examples of trust-building behavior at Amistad • Individual and group ranking of culture aspects with stated justification
April 2023	1. Describe the characteristics of authoritative leadership and, using a video example, distinguish exaggerated portrayals from realistic practice and state when the style is warranted. 2. Analyze a prior workplace conflict they would have handled differently and identify what they would change. 3. Evaluate the five conflict-resolution suggestions and the Myatt commentary, and select the most important for their own role.	• Authoritative leadership video • Conflict management video • Recommended reading on conflict resolution • Mike Myatt quotation on leadership and conflict (Forbes)	• Shout-out on characteristics of democratic leadership • Structured video critique • Roundtable analysis of a real prior conflict and of an Amistad conflict • Stated action items (one or two learnings to carry forward)
May 2023	1. Describe the characteristics of laissez-faire leadership and weigh its potential benefits against its potential negative effects. 2. Analyze a conflict observed at Amistad in the past month, including the issue, how it was resolved, and whether a better approach was available. 3. Explain how emotional intelligence contributes to leadership, including the relationship between empathy and social awareness.	• Laissez-faire leadership video • Harvard Business School Online article and video on emotional intelligence in leadership • Marshall Rosenberg quotation on responsibility for behavior, thought, and feeling • Supplementary leadership video	• Shout-out on characteristics of authoritative leadership • Guided video discussion • Roundtable on an Amistad example of strong or poor emotional intelligence and its outcome
June 2023	1. Describe the characteristics of transactional leadership and identify where it is and is not effective at Amistad. 2. Reflect on an occasion in the past month of communicating poorly with an employee and identify the cause of the resulting conflict. 3. Give examples of effective relationship management at Amistad and explain why emotional intelligence matters in a leadership role.	• Transactional leadership video • Harvard Business School Online article and video on emotional intelligence in leadership • Marshall Rosenberg quotation • Supplementary leadership video	• Shout-out on characteristics of laissez-faire leadership • Guided video discussion on effectiveness in context • Roundtable self-reflection and identification of Amistad examples
July 2023	1. Describe the characteristics of coaching leadership and identify where the approach is and is not effective at Amistad. 2. Distinguish the characteristics of high and low emotional intelligence. 3. Apply seven management recommendations—acting as a manager rather than a friend, dealing consistently with employees, accepting final accountability, focusing on outcome rather than process, delegating, not overstating one's own knowledge, and reconciling theory with real-world practice—to their own supervisory work.	• Coaching leadership video • Key leadership recommendations video (first 7 minutes)	• Shout-out on characteristics of transactional leadership • Roundtable identification of high and low emotional intelligence characteristics • Discussion of the seven recommendations against current practice
August 2023	1. Describe the characteristics of pacesetter leadership and identify where the approach is and is not effective at Amistad. 2. Give an example from the past month in which final accountability rested with them. 3. Apply a second set of recommendations—declining requests when appropriate, sharing a vision, being open with people, favoring action over inaction, under-promising and over-delivering, documenting agreements, and not asking of others what one would not do oneself—and reflect on the distinction between managers who make excuses and leaders who do not.	• Pacesetter leadership video (first 2 minutes) • Key leadership recommendations video (first 7 minutes) • Written reflection prompt on excuse-making versus leadership	• Shout-out on characteristics of coaching leadership • Roundtable example of accountability in the preceding month • Individual reflection shared with the group
October 2023	1. Compare the six leadership styles covered across the series and select an appropriate style for a given clinical or administrative situation. 2. Give an example of a recent situation in which the recommendation to document agreements applied. 3. Demonstrate leadership and communication skills in four simulated scenarios: a request for paid time off, disrespect by a coworker, anger directed at management, and a tardy or absent employee.	• Consolidated review of the six leadership styles • Closing video on leading and inspiring, with reflection on application at Amistad • Four role-play scenario briefs	• Shout-out on characteristics of pacesetter leadership • Roundtable example of applying the documentation recommendation • Facilitator and peer observation of role-play performance across four scenarios

Appendix 4: Survey instrument

The post-intervention survey was developed by the program facilitators (U.B., E.B.) on the basis of the program learning objectives using Google Forms. It was not derived from a previously published instrument, and it was not formally validated or pilot-tested prior to administration. The instrument was administered anonymously and online in June 2024, following completion of the 13-month program, and was redistributed once, three weeks later, to maximize participation.

The instrument comprised three components: (i) participant background and demographic items; (ii) quantitative items rated on five-point Likert scales, comprising six agreement items scored 1 (not at all) to 5 (completely) and two quality items - training format and delivery, and trainer effectiveness - scored 1 (very poor) to 5 (excellent); and (iii) open-ended free-text items inviting general feedback and suggestions for program improvement.

The complete instrument, reproduced verbatim as administered, appears below:

Post-Series Participant Evaluation, CHC Leadership Development Program, Amistad Health

1. Position or Role at Amistad Health

Open-ended response (free text).

2. Type of Patients you care for in Amistad Health

Single selection.

A. Pediatric

B. Adult

C. Both

3. Years of Clinical Experience

Single selection.

A. 1-5 years

B. 5-10 years

C. 10-20 years

D. > 20 years

4. Years of Leadership Experience

Single selection.

A. 1-5 years

B. 5-10 years

C. 10-20 years

D. > 20 years

Items 5-10 were rated on a five-point scale anchored at 1 = Not at all and 5 = Completely.

5. The leadership series met my expectations.

Five-point scale: 1 = Not at all; 2; 3; 4; 5 = Completely.

6. The leadership series was engaging.

Five-point scale: 1 = Not at all; 2; 3; 4; 5 = Completely.

7. I was satisfied with the content covered in leadership sessions.

Five-point scale: 1 = Not at all; 2; 3; 4; 5 = Completely.

8. The topics covered aligned with my professional responsibilities.

Five-point scale: 1 = Not at all; 2; 3; 4; 5 = Completely.

9. The sessions were effective in enhancing my understanding of healthcare leadership concepts.

Five-point scale: 1 = Not at all; 2; 3; 4; 5 = Completely.

10. I feel more prepared to apply leadership principles in my daily work after attending these sessions.

Five-point scale: 1 = Not at all; 2; 3; 4; 5 = Completely.

Items 11-12 were rated on a five-point scale anchored at 1 = Very Poor and 5 = Excellent.

11. My rating for the format and delivery of the training materials (e.g., presentations, exercises, case studies) is:

Five-point scale: 1 = Very Poor; 2; 3; 4; 5 = Excellent.

12. My rating for the effectiveness of the trainer/facilitator is:

Five-point scale: 1 = Very Poor; 2; 3; 4; 5 = Excellent.

13. Please provide any suggestions or comments you have to help us improve future healthcare leadership training programs

Open-ended response (free text).

Appendix 5: Initial needs assessment questionnaire

Overview

Prior to the commencement of the leadership development sessions, an initial needs assessment was distributed to participants. The objective of this assessment was to identify baseline leadership competencies, self-perceived areas for growth, and specific organizational support requirements.

Administration and Data Privacy

Participants were provided with a structured questionnaire and instructed to complete it prior to the inaugural discussion. Responses were collected directly by the primary facilitator. The raw data were kept strictly confidential; responses were subsequently aggregated, anonymized, and synthesized to guide meaningful group discussions without compromising individual privacy.

Questionnaire: The following open-ended questions were administered to the review group:

1. What is your best leadership quality?

2. What leadership quality would you like to be better at?

3. What is the thing about leadership you feel you know the least about?

4. What is your biggest struggle in supervising others?

5. What are others’ biggest struggles in working with you?

6. What is the most important thing Amistad can do for your development?

Appendix 6: Online educational materials

Overview

The leadership development curriculum incorporated selected online educational materials to supplement facilitated discussions and reinforce session-specific learning objectives. Materials included leadership-style videos, organizational-culture resources, conflict-management instruction, emotional-intelligence content, and leadership-recommendation videos. These resources supported participant reflection, group discussion, and application of concepts to workplace scenarios across the monthly sessions.

**Table 7:** Online materials.

Month/session	Video/article	Link
February 2023	Leadership video: Simon Sinek talk on culture, trust, cooperation, and “leaders eat last”	https://www.youtube.com/watch?v=lmyZMtPVodo
March 2023	Workplace-culture video: 6 Essential Aspects of Workplace Culture	https://www.youtube.com/watch?v=f98WUMHGw4
April 2023	Leadership-style video: Authoritative leadership	https://www.youtube.com/watch?v=L6wgfUypUuM
	Conflict-management video	https://www.youtube.com/watch?v=aSq5IMpQReM
May 2023	Leadership-style video: Laissez-faire leadership	https://www.youtube.com/watch?v=fqYSWzb62Gs
	Online article by Simon Sinek: Emotional Intelligence in Leadership	https://online.hbs.edu/blog/post/emotional-intelligence-in-leadership
June 2023	Leadership-style video: Transactional leadership	https://www.youtube.com/watch?v=6xxwqv9gPxc
	Online article/learning resource: Emotional Intelligence in Leadership	https://online.hbs.edu/blog/post/emotional-intelligence-in-leadership
July 2023	Leadership-recommendations video, first 7 minutes	https://www.youtube.com/watch?v=1tlw5E8XjOw
August 2023	Leadership-style video: Pacesetter leadership, first 2 minutes	https://www.youtube.com/watch?v=amJ-srPS9wg
	Leadership-recommendations video, first 7 minutes	https://www.youtube.com/watch?v=1tlw5E8XjOw
October 2023	Leadership/inspiration video: “Leading, inspiring to the world ahead”	https://www.youtube.com/watch?v=Vwbkz0DSkHU
